# Metalloproteinases and their Inhibitors under the Course of Immunostimulation by CPG-ODN and Specific Antigen Inhalation in Equine Asthma

**DOI:** 10.1155/2019/7845623

**Published:** 2019-06-17

**Authors:** Ann Kristin Barton, Tarek Shety, John Klier, Sabine Geis, Ralf Einspanier, Heidrun Gehlen

**Affiliations:** ^1^Equine Clinic, Freie Universitaet Berlin, Germany; ^2^Animal Medicine Department, Faculty of Veterinary Medicine, Zagazig University, Zagazig, Egypt; ^3^Centre for Clinical Veterinary Medicine, Equine Clinic, LMU Munich, Germany; ^4^Institute of Veterinary Biochemistry, Freie Universitaet Berlin, Germany

## Abstract

**Objectives:**

Inhalation of immunostimulatory bacterial DNA segments (cytosine-phosphate-guanosine-oligodeoxynucleotides, CpG-ODN) normalizes clinical and cytologic parameters in severe equine asthma. We hypothesized that CpG-ODN inhalation also reduces the misbalance of elastinolytic activity in asthmatic horses.

**Methods:**

Twenty asthmatic horses diagnosed by clinical examinations using a scoring system were included. All horses inhaled CpG-ODNs for 14 days in 2-day intervals. Matrix metalloproteinase (MMP-2/-9) and tissue inhibitors of metalloproteinase (TIMP-1/-2) concentrations were measured in tracheal aspirates using equine sandwich ELISAs before and 2 and 6 weeks after CpG-ODN inhalation.

**Results:**

MMP and TIMP concentrations correlated with the results of clinical scoring in all stages of equine asthma. Inhalation therapy led to significant reductions in clinical scores. MMP-2, MMP-9, and TIMP-2 concentrations were significantly reduced immediately, and all MMP and TIMP concentrations 6 weeks after therapy.

**Discussion:**

In equine asthma, overexpression of MMPs contributes to pathological tissue destruction, while TIMPs counteract MMPs with overexpression leading to fibrosis formation. The results of this study show that CpG-ODN inhalation may be an effective therapy to address a misbalance in equine asthma.

**Conclusions:**

Misbalance of elastinolytic activity seems to improve by CpG-ODN inhalation for at least 6 weeks posttherapy, which may reduce the remodeling of the extracellular matrix. Further studies should evaluate this effect in comparison to glucocorticoid inhalation therapy.

**Significance:**

CpG-ODN inhalation may be an effective therapy in the prevention of pulmonary fibrosis formation in equine asthma.

## 1. Introduction

Although environmental dust reduction remains the cornerstone in equine asthma therapy [1], drug therapy may also be indicated, both in situations where the implementation of appropriate environmental changes is problematic and in horses with severe clinical disease, as a necessary adjunct to the implementation of optimal environmental changes. Unfortunately, despite glucocorticoids and bronchodilators suppressing the inflammatory response and ameliorating clinical signs of bronchial obstruction, they are not curative. A new therapeutic causative approach for equine asthma is inhalation of gelatinase particle bound cytosine-phosphate-guanosine-oligodeoxynucleotides (CpG-ODN) as described by Klier et al. [2–5]. The CpG motive, a distinct sequence of nucleotides appearing recurrently in bacterial and viral DNA, contains a central cytosine-phosphate-guanosine-dinucleotide. These CpG sequences are common in prokaryotic DNA but are rare and commonly suppressed in mammalian DNA. In addition, they are usually methylated in mammals, while they are unmethylated in viral and bacterial DNA. These unmethylated CpG motives are recognized as danger signals in many species explaining their immune-stimulatory effect. Within the cell, the unmethylated DNA motives are recognized as pathogen-associated molecular patterns (PAMPs) by the intracellular toll-like receptor 9 (TLR 9) and lead to a strong Th1 immune response, which would be appropriate for a viral, bacterial, or parasite infection [6, 7]. In the case of equine asthma, this leads to an immune shift from a Th2 to a Th1 reaction, suppression of IL-4, increase in IL-10 and IFN-gamma, and a cytological reduction in neutrophils in respiratory secretions [2, 3]. In several studies, the authors could show an improvement in clinical signs, respiratory secretion cytology, and arterial blood gas analysis in horses suffering from severe equine asthma.

Remodeling of the extracellular matrix (ECM) of pulmonary connective tissue is a continuous process allowing growth and regeneration. To allow for healing, growth, and maintenance of tissue stability, a balance exists between degradation. Zinc-dependent endopeptidases, so-called matrix metalloproteinases (MMPs), are the most important proteolytic enzymes, and resynthesis of extracellular matrix structures in healthy subjects [8]. Several studies have demonstrated a central role of MMPs in chronic respiratory disease in human asthma and COPD as well as equine asthma [9–13]. In the airways of asthmatic patients, activated fibroblasts account for an excessive matrix production. This bronchial remodeling is also seen in equine asthma [14]. An imbalance between different MMPs, particularly MMP-9, and their tissue inhibitors (TIMPs), particularly TIMP-1, which is the most widely distributed and acts on all active MMPs, has been shown in several studies. Increased levels of MMP-9 [15, 16] and also MMP-2 [17, 18] as well as elevated TIMP-1 and TIMP-2 levels are found in the airways of asthmatic patients [15, 17–19]. This suggests that pathological airway remodeling in asthma, resulting in airway fibrosis, may be a consequence of overrepair mechanisms. MMPs degrade the ECM directly, but this may counteract fibrosis formation [20]. However, an excessive degradation over a longer period of time may also result in a feedback of overrepair cycles, leading to increased synthesis and deposition of ECM [21].

In former studies of our group, we could show a misbalance in elastinolytic and collagenolytic activity in equine asthma, which may contribute to fibrosis formation in long-term disease [13, 22]. Increased concentrations of MMPs and TIMPs were found in bronchoalveolar lavage fluid (BALF) of asthmatic horses suffering from mild-to-moderate as well as severe disease.

As the complex inflammatory processes in equine asthma have been studied intensely, but are still not fully understood [1, 23], we hypothesized that CpG-ODN inhalation might also affect the elastinolytic processes within the ECM contributing to bronchial remodeling and that pulmonary fibrosis formation might be inhibited by inhalation of CpG-ODN as a consequence of a downregulation of the underlying allergic inflammation. The objectives of the present study were to compare the concentrations of MMP-2 and MMP-9 as well as TIMP-1 and TIMP-2 in tracheal aspirate (TA) with clinical findings and cytology results under the course of CpG-ODN inhalation.

## 2. Material and Methods

In the present study, 10 horses inhaled CpG-ODN and 10 horses inhaled CpG-ODN in addition to specific allergens; samples were obtained from a prior study by our group [2]. As the addition of specific allergens did not have an impact on the effect of CpG-ODN inhalation, we included all samples as a study population to increase the statistical power concerning the differences in MMP and TIMP concentrations in tracheal wash samples at three time points, before and 2 weeks and 6 weeks after inhalation therapy.

### 2.1. Preparticipation Examination

A total of 20 horses of mixed breeds were presented for participation in this prospective clinical trial with a history of equine asthma. The study was approved by the regional legal agency for animal experiments of the Government of Upper Bavaria, Germany (No. 55.2-1-54-2531-31-10). The owners gave permission to involve their horses in the study. The preparticipation examination included respiratory rate at rest, breathing type, auscultation of the trachea and both lung fields, endoscopy and cytology of tracheal aspirates, arterial blood gas analysis, and intrapleural pressure measurement. Inclusion criteria were tachypnea (>16/min), increased abdominal effort in expiration, neutrophilia >25% in cytology, hypoxia at rest (partial oxygen pressure< 90 mmHg), and increased intrapleural pressure (>15 cm H_2_O). Results were included in a scoring system modified from prior studies [24] as shown in Table 1 to classify the horses as mildly, moderately, or severely asthmatic.

All examinations and inhalation therapy took place at the horses' customary stables. During the study period, no changes in the management of the horses were implemented. In addition, none of the horses had received any additional medications for at least 8 weeks before the start and throughout the duration of the study.

### 2.2. CpG-ODN Inhalation

Nanoparticles were produced and loaded with CpG-ODN 2216 (Biomers GmbH, Ulm, Germany) as previously described [2, 3]. Each inhalation utilized 187.5 *μ*g CpG-ODN, bound to 3.75 mg GNP and dispersed in 2.5 ml highly purified water (HPW), with a final concentration of 1.5 mg/ml GNP and 0.075 mg/ml CpG-ODN. The inhalation regimen was conducted with Equine Haler®™ (Equine HealthCare Aps, Hoersholm, Denmark) and Aeroneb® Go micropump nebulizer (Aerogen, Galway, Ireland) as previously described [2, 3]. The seven inhalations in each horse were administered every other day.

### 2.3. Clinical Scoring and Clinical Pathology

The first evaluation of the horses occurred before the treatment (*t*_0_) and included clinical evaluation, bronchoscopy, and evaluation of pulmonary function parameters, as well as cytological, immunological, and laboratory-chemical parameters. The second evaluation (*t*_1_) occurred after the completion of the seventh inhalation 2 weeks later. The third evaluation (*t*_2_) occurred 6 weeks after the last treatment, in order to determine any long-term effect.

### 2.4. Laboratory Analysis of Interleukins, MMPs, and TIMPs

Venous blood samples taken from the jugular vein and 20 ml of tracheal wash fluid were centrifuged, and the supernatant was immediately frozen using liquid nitrogen and stored at −80°C until assayed. Protein concentrations were measured and used for normalization. Concentrations of the cytokines IL-4, IL-10, IL-17, and IFN-*γ* were measured in tracheal wash fluid, using equine sandwich ELISAs (Equine IL-4 kit, Equine IL-10 kit, Equine IL-17 kit, and Equine IFN-*γ* kit; R&D Systems, Minneapolis, USA) found to be reliable in prior studies by our group [5] and following the manufacturer's protocol. MMP-2 and MMP-9 as well as TIMP-1 and TIMP-2 were also measured in BALF using sandwich ELISAs (Equine MMP-2 kit, Equine MMP-9 kit, Equine TIMP-1 kit, and Equine TIMP-2 kit; USCN Life Science Inc., China) used in former studies by our group [13, 22]. MMP and TIMP concentrations were not evaluated in serum, as previous studies had shown no significant changes in different stages of equine asthma (data not published). For all parameters evaluated, standards and samples were set up in duplicates and the absorbance was measured with an ELISA microplate reader at 450 nm immediately.

### 2.5. Statistics

Data were statistically analyzed using SPSS and were expressed as mean ± standard deviation (SD). The data were tested for normal distribution using the Shapiro-Wilk Test. While some data were found to be normally distributed, other was found not to, so we preferred nonparametric tests for the whole data. The level of significance was set at *P* < 0.05. The Kruskal-Wallis *H* test was used to compare between the severities of different disease group (mild, moderate, and severe equine asthma) followed by post hoc testing using the Mann–Whitney *U* test for a 2-group comparison to determine intergroup differences. Correlations between neutrophil percentages and the concentrations of MMP-2, MMP-9, TIMP-1, and TIMP-2 were calculated using the Spearman correlation test.

## 3. Results

The results of the clinical examinations and interleukin measurements have been published in a former paper [2]. They are summarized here to allow the later discussion in correlation to MMPs and TIMPs.

### 3.1. Clinical Scoring

In all 20 horses presented for participation in this study with a history of equine asthma, the disease was confirmed. Animals were classified as mild (*n* = 4), moderate (*n* = 11), or severe equine asthma (*n* = 5) at the beginning of the study. As described in the former paper, scores for respiratory rate, breathing type, auscultation, nasal discharge, AaDO_2_, PaO_2_, amount, and viscosity of respiratory secretions were all reduced significantly 6 weeks after CpG-ODN inhalation therapy [2]. The latest three were already reduced significantly after 2 weeks.

### 3.2. Interferon-*γ*, Interleukin-4, Interleukin-10, and Interleukin-17

As described by Klier et al. [2], IL-4 concentration showed a significant decrease in tracheal wash fluid 2 and 6 weeks after the end of CPG-ODN inhalation therapy and in serum after 6 weeks. Concentrations of IL-10 and IL-17 did not decrease, neither in tracheal wash (IL-10 and IL-17) nor in serum (IL-10). IFN-*γ* concentration was reduced after 2 and 6 weeks in serum, while no decrease was found for tracheal wash [2].

### 3.3. MMP-2 Elisa

Significant increases in MMP-2 concentration in tracheal wash were found between different severity grades of equine asthma rising from 14.0 ± 0.2 ng/ml in mildly to 18.4 ± 0.3 ng/ml in severely affected horses. Under CpG-ODN inhalation therapy, overall MMP-2 concentration decreased from 15.9 ± 0.5 ng/mg (before therapy), over 14.6 ± 0.3 ng/ml (2 weeks after therapy), to 13.0 ± 0.3 ng/ml (6 weeks after therapy). This decrease was significant 2 weeks after therapy.

### 3.4. MMP-9 Elisa

More obvious differences between disease severity groups were detected for MMP-9 in tracheal wash rising from 280.2 ± 94.0 in mildly to 933.8 ± 20.0 pg/ml in severely asthmatic horses. Under CpG-ODN inhalation therapy, overall MMP-9 concentration decreased from 606.9 ± 85.5 pg/mg (before therapy), over 324.8 ± 81.7 pg/ml (2 weeks after therapy), to 210.6 ± 38.4 pg/ml (6 weeks after therapy). This decrease was significant 6 weeks after therapy.

### 3.5. TIMP-1 Elisa

Significant increases in tracheal wash concentration between different severity grades of equine asthma were found for TIMP-1 rising from 93.6 ± 12.6 ng/ml in mildly to 179.7 ± 6.4 ng/ml in severely affected horses. Under CpG-ODN inhalation therapy, overall TIMP-1 concentration decreased from 139.4 ± 11.3 ng/mg (before therapy), over 115.4 ± 15.9 ng/ml (2 weeks after therapy), to 88.4 ± 16.9 ng/ml (6 weeks after therapy). This decrease was significant 6 weeks after therapy.

### 3.6. TIMP-2 Elisa

Significant increases in tracheal wash TIMP-2 concentration between different severity grades of equine asthma were found rising from 99.3 ± 13.8 ng/ml in mildly to 169.5 ± 6.8 ng/ml in severely affected horses. Under CpG-ODN inhalation therapy, overall TIMP-2 concentration decreased from 124.6 ± 10.6 ng/mg (before therapy), over 77.6 ± 5.3 ng/ml (2 weeks after therapy), to 70.1 ± 6.3 ng/ml (6 weeks after therapy). This decrease was significant 2 weeks after therapy.

Spearman correlation test showed that there were positive correlation of neutrophils' percentages in tracheal wash cytology with MMP-2 and MMP-9 concentrations (*r* = 0.612 and *r* = 0.651, respectively) and the correlation was significant at *P* < 0.05. There were also positive correlations of neutrophils' percentages with TIMP-1 and TIMP-2 concentrations (*r* = 0.708 and *r* = 0.667, respectively), and the correlation was significant at *P* < 0.01.

Overall, CPG-ODN inhalation therapy led to significant reductions in clinical scores; concentrations of IL-4, MMP-2, MMP-9, and TIMP-2 in tracheal wash decreased after 2 weeks, and all MMP and TIMP concentrations 6 weeks after therapy. A concluding summary of all MMP and TIMP measurements is presented in Tables 2 and 3.

## 4. Discussion

To our knowledge, this is the first study to show that the misbalance of elastinolytic activity of the extracellular matrix in equine asthma might be positively influenced by CpG-ODN inhalation for at least 6 weeks posttherapy. If this is actually the case, the remodeling of the extracellular matrix and fibrosis formation might be reduced in this common disease. In human asthma, the degree of MMP activity can be linked to the intensity of the inflammatory processes in the airways. The MMP/TIMP balance or rather disbalance is widely accepted to have a role in the pathogenesis of airflow limitation and reflect the extent of structural changes in the lung [20, 25, 26].

In severe equine asthma, increased MMP-2 and MMP-9 levels were shown using gelatin zymography [9]. Particularly, MMP-9 levels were clearly upregulated in different stages of equine asthma [13]. Both MMP-2 and MMP-9 showed a correlation to stable dust concentrations [27–29]. MMP-9 levels decreased with BALF neutrophilia under budesonide, an inhalative glucocorticoid [22]. This was not the case for MMP-2, which seems to be more of a housekeeping enzyme [10]. Other authors also showed increased activities of elastinolytic MMP-9 and MMP-13 as well as collagenolytic MMP-8 in equine asthma [11, 12].

Tissue inhibitors of metalloproteinases (TIMPs) are natural antagonists of MMPs [30, 31]. In human COPD, MMP-9 and TIMP-1 concentrations in BALF increased, which was not the case in plasma [32]. TIMP-1 and TIMP-2 concentrations as well as MMP-TIMP ratios have also been studied in equine asthma [13] showing increased levels of TIMPs in mild-to-moderate as well as severe equine asthma compared to controls and normalization of MMP-TIMP ratios under budesonide therapy [22].

In unison with former studies, MMP-2/-9 and TIMP-1/-2 levels increased with severity of equine asthma and all MMPs/TIMP concentrations decreased with clinical score parameters within 2-6 weeks after CPG-ODN inhalation. Interestingly, this effect was not yet found at *t*_1_ (2 weeks postinhalation), but at *t*_2_ (6 weeks post inhalation) for MMP-9 and TIMP-1, which are probably the most important enzymes in natural disease. This shows that CpG-ODN does not only have a long-term effect, but that therapy of extracellular remodeling may even require longer terms of treatment. This theory should be substantiated by longitudinal studies including lung tissue biopsies to look at fibrosis formation in long-term disease. A possible long-term effect should be further evaluated and tried to be extended, possibly by increasing the numbers of inhalations, increasing the time-span of inhalation intervals or repetition of inhalation therapy after some weeks or even months. Remodeling of the ECM and fibrosis formation is a very slow process in human and equine asthma; therefore, models of long-term therapy should be developed.

The observed positive effect of CpG-ODN inhalation therapy on clinical parameters and cytology of respiratory secretions has been confirmed in several in vitro and in vivo studies for equine asthma and could be shown to last at least 6 weeks [2–5, 33]. All clinical studies were field studies; therefore, the combined effect of CpG inhalation and a low-dust environment remains to be studied. It is well known that clinical parameters, cytology, and lung function after common pharmacological therapy in equine asthma including systemic or inhalative glucocorticoid therapy, secretolytics, and bronchodilators improve much better under adjacent measures to reduce the allergen and dust-particle load in the environment [1, 14, 23]. In this study, horses were kept in their home stables in different environments and no environmental changes were implemented throughout the study. All included subjects had clinical and cytological evidence of airway obstruction and inflammation, showing they were not in disease remission. On the other hand, this allows for a better evaluation under natural conditions of the effect of CpG-ODNs on the elastinolytic activity, as implementation of a low-dust environment may have also led to decreasing inflammation and MMP/TIMP concentrations in tracheal aspirates. Nevertheless, further studies should include a control group inhaling placebo to study the effect of seasonal changes on elastinolytic activity. Although reference values of MMP and TIMP concentrations have only been described in BALF in healthy controls [13], we assume that MMP and TIMP levels in tracheal wash samples were elevated, as former studies have shown a strong correlation between their concentrations in BALF and clinical and cytological parameters. Nevertheless, the authors suggest that further prospective studies should rather use BALF, as proposed by current literature for the diagnoses of all stages of equine asthma and used in prior studies on MMP/TIMP misbalance.

The relation between clinical improvement and decreasing MMP/TIMP levels under CpG-ODN inhalation might not be directly causative but reflect the degree of decreasing airway inflammation and achievement of disease remission. The direct mechanism, by which CpG-ODN inhalation positively influences the misbalance in elastinolytic activity in equine asthma, needs further studies. Former studies have shown that several interleukins (IL-4, IL-10, and IFN-*γ*) may have to be considered, as their levels changed in accordance with the Th2/Th-1shift [3, 5], and in this study, a decrease was also found, at least for IL-4 (serum and tracheal wash) and IFN-*γ* (serum). A downregulation of the Th2 immune response and thus the IL-4 secretion is expected by the agonistic activity of CpG-ODNs on the intracellular TLR9 [2, 34, 35]. Decreasing INF-*γ* concentrations in serum over 6 weeks show a long-lasting anti-inflammatory effect of CpG-ODN inhalation as well [36–38]. The high positive correlations of MMPs and neutrophil percentages in BALF suggest these cells to be the origin of MMPs, in particular MMP-9, in equine asthma with neutrophils being one of the most important sources of MMPs [13]. This is supported by the high correlations between MMP and TIMP concentrations and neutrophil percentages in tracheal aspirate cytology in the presented study. On the other hand, IL-4 and INF-*γ* concentrations also correlate with decreasing airway neutrophilia [2], which may be possible cross-links, but as equine asthma pathophysiology is so complex, further mechanisms may contribute to the positive influence of CpG-ODNs on elastinolytic activity and remodeling of the extracellular matrix.

In conclusion, this study shows further positive effects of CpG-ODN inhalation in equine asthma possibly improving the elastinolytic activity for at least several weeks, which may reduce the remodeling of the extracellular matrix. Further studies should evaluate this effect in comparison to glucocorticoid inhalation therapy and with adjacent improvement of the environment in terms of allergen and dust-particle load.

## Figures and Tables

**Table 1:** Clinical score modified from Barton et al. [24].

		Score	Max. points
(1) Cough induction	No cough after manual compression of larynx	0	1
	Coughing during manual larynx compression	1	
	Very frequent coughing	1	
	Spontaneous coughing	1	

(2) Dyspnoea at rest	Prolonged expiration	1	3
	Increased abdominal effort in expiration	1	
	Sinking of the intercostal area	3	
	Nostril flare	3	
	Heaves line	3	
	Anal pumping	3	

(3) Respiratory rate	8-16/min	0	2
	17-23/min	1	
	≥24/min	2	

(4) Lung auscultation	Rattling	2	2
	Crackle	2	
	Wheezing	2	

(5) Tracheobronchoscopy	Significantly increased secretions with moderate viscosity	1	2
	Highly increased secretions with high viscosity	2	
	Thickened carina of the trachea	1	

(6) Cytology tracheal aspirate	Neutrophils <8%	0	3
	Neutrophils 8-15%	1	
	Neutrophils 15-25%	2	
	Neutrophils >25%	3	

(7) Arterial blood gas	AaDo_2_: 0-7 mmHg	0	2
	AaDo_2_: 7-14 mmHg	1	
	AaDo_2_: >14 mmHg	2	

≤6 points: mild asthma, 6-10 points: moderate asthma, 11-15 points: severe asthma.

**Table 2:** Concentrations of MMP-2, MMP-9, TIMP-1, and TIMP-2 in tracheal aspirates from horses suffering from equine asthma of different disease severity (mild, moderate, and severe).

	Mild asthma (*n* = 4)	Moderate asthma (*n* = 11)	Severe asthma (*n* = 5)
MMP-2 (ng/ml)	14.0 ± 0.2 14.05 (13.5 - 14.4)	15.6 ± 0.2^∗^ 15.43 (15.1 - 16.7)	18.4 ± 0.3^∗^ 18.38 (17.9 - 19.1)

MMP-9 (pg/ml)	280.2 ± 94.1 271.89 (107.3 - 469.7)	535.6 ± 104.8 536.97 (178.5 - 819.1)	933.8 ± 20.0^∗^ 916.77 (908.3 - 993.2)

TIMP-1 (ng/ml)	93.6 ± 12.6 104.81 (56.0 – 108.7)	144.5 ± 9.3^∗^ 152.36 (103.3 – 163.1)	179.7 ± 6.4^∗^ 181.45 (165.0 – 191.0)

TIMP-2 (ng/ml)	99.3 ± 13.8 101.07 (65.6 - 129.4)	102.2 ± 11.9 104.22 (68.2 - 137.1)	169.5 ± 6.7^∗^ 165.99 (157.3 - 188.8)

The results are expressed as mean ± SE, median, and range. The statistical analysis was performed by the Kruskal-Wallis *H* test, and ∗ indicates significant differences between different severity groups.

**Table 3:** Concentrations of MMP-2, MMP-9, TIMP-1, and TIMP-2 in tracheal aspirates from 20 asthmatic horses measured on admission (*t*_0_), 2 weeks (*t*_1_), and 6 weeks (*t*_2_) after CpG-ODN inhalation.

	*t*_0_	*t*_1_	*t*_2_
MMP-2 (ng/ml)	15.9 ± 0.5	14.6 ± 0.3^∗^	13.0 ± 0.3^∗^
MMP-9 (pg/ml)	606.9 ± 85.5	324.8 ± 81.7	210.6 ± 38.4^∗^
TIMP-1 (ng/ml)	139.4 ± 11.3	115.4 ± 15.9	88.4 ± 16.9^∗^
TIMP-2 (ng/ml)	124.6 ± 10.6	77.6 ± 5.3^∗^	70.1 ± 6.3^∗^

The results are expressed as mean ± SE (min.-max.). ∗ marks a significant reduction of MMP/TIMP concentration in comparison to *t*_0_.

## Data Availability

The data used to support the findings of this study are available from the corresponding author upon request.
